# Does More Education Always Lead to Better Health? Evidence from Rural Malaysia

**DOI:** 10.1155/2015/539212

**Published:** 2015-01-05

**Authors:** Gareth Leeves, Ireneous Soyiri

**Affiliations:** ^1^School of Business, Monash University Malaysia, Jalan Lagoon Selatan, 47500 Bandar Sunway, Selangor, Malaysia; ^2^South East Asia Community Observatory (SEACO), Jeffrey Cheah School of Medicine and Health Sciences, Monash University Malaysia, 146 Jalan Sia Her Yam, 85000 Segamat, Johor, Malaysia

## Abstract

*Background*. Education is usually associated with improvement in health; there is evidence that this may not be the case if education is not fully utilised at work. This study examines the relationship between education level, occupation, and health outcomes of individuals in rural Malaysia.* Results*. The study finds that the incidence of chronic diseases and high blood pressure are higher for tertiary educated individuals in agriculture and construction occupations. This brings these individuals into more frequent contact with the health system. These occupations are marked with generally lower levels of education and contain fewer individuals with higher levels of education.* Conclusions*. Education is not always associated with better health outcomes. In certain occupations, greater education seems related to increased chronic disease and contact with the health system, which is the case for workers in agriculture in rural Malaysia. Agriculture is the largest sector of employment in rural Malaysia but with relatively few educated individuals. For the maintenance and sustainability of productivity in this key rural industry, health monitoring and job enrichment policies should be encouraged by government agencies to be part of the agenda for employers in these sectors.

## 1. Introduction

It is usually accepted that better educated individuals will have better health outcomes. We know that higher education is associated with lower mortality rates; and Kulhánová et al. provide a recent summary of evidence on this issue [1]. Education also impacts social and mental function in later life [2]. The effect of education on health in later life can occur through a number of channels. Namely, higher income and socioeconomic status are associated with better health related quality of life (HRQL) in the USA (though not in Canada) [3, 4]. Similar results have been obtained for European countries [5].

More pertinent to the present study is the contribution by Valenzuela and Sachdev who found that mentally demanding work and the level of stimulation in the job are influential for subsequent cognitive status [6]. People in lower skilled occupations are more at risk of cognitive impairment [7]. In sum, the evidence suggests that education's role in generating higher income and access to more stimulating and higher status jobs produces health benefits in later life.

However, the relationship between education and work satisfaction for an individual is more complex. There are links between the level of education of an individual, the requirements of their job, and their job satisfaction. In particular, the overeducated, those working in jobs that do not require skills commensurating with their qualifications, are less satisfied than other workers [8–11]. The reduction in satisfaction includes both their work (job satisfaction) and general wellbeing (life satisfaction). Job satisfaction is related to health. A comprehensive meta-analysis of 485 predominantly cross-sectional studies with measures of both job satisfaction and health shows an overall (simple) correlation across all health measures of 0.312 [12]. The strongest correlations were found amongst job satisfaction and self-reported measures of health, depression, self-esteem, and so forth. Attempts to reveal a relationship between more objective measures of physical health and job satisfaction have been less fruitful [13]. More recent research [14] suggests that increased job satisfaction does reduce reported incidents of doctor visits and impediments like being able to climb stairs and appears to reduce events like workers classifying themselves as disabled. It is therefore expected that individuals not utilizing their education at work will display more signs of adverse health outcomes, due to the link between job satisfaction and health outcomes. Hence, the expected benefits of education in later life, detailed earlier, may not eventuate when education is underutilized.

The focus of this study is on education work and health in a rural area of the developing country of Malaysia. Little is known about the relationship between education, work, and health outcomes in rural setting within a developing country. One recent study of households in Fiji found that Indo-Fijians had better health outcomes than native Fijians. This was linked to higher education levels which generated higher incomes [4]. Utilizing education may be more difficult in a rural environment where employment opportunities are more restricted. A recent study of the cognitive status of elderly Malays in rural Malaysia noted that occupation could be a significant determinant of cognitive impairment in later life [15]. This study is the first to provide evidence of the relationship between education, employment, and health outcomes in rural Malaysia. We investigate education level and health outcomes in differing occupations. Given the data constraints, any correlation found between education levels and worsening health outcomes could be linked to a number of contributing factors including stress and lack of job satisfaction; some of these will be investigated in further research as more waves of data become available. Nevertheless, we can identify how increased education levels manifest themselves in significant health outcomes and frequency of contacts with the health system in a rural setting. This can aid the development of strategies that might bring benefits later in the individual's life.

Workers health and wellbeing in rural locations are of particular importance given the ageing populations in these areas, where social networks and health support services can be more limited [16]. In this paper we report the relationship between individuals with higher education in different occupations and health outcomes. We use statistical inference drawn from differing regression methods that are detailed in the following section, which are shown to be appropriate given the nature of the data contained in our survey. In particular we seek to address the following research questions: (a) whether there is an inverse relationship between the level of education and health outcomes in occupations where education levels are low and (b) whether there is a positive correlation between education level and contacts with the health system in these occupations.

## 2. Materials and Methods

The data used is drawn from the first wave of the South East Asian Community Observatory (SEACO) survey of households in the Segamat district of Johor. This work is conducted as one of a number of Demographic and Health Surveillance Sites (DHSS) for the collection of longitudinal data on a fully enumerated population within a circumscribed geographical location [17]. The dataset covers the entire population of rural towns and villages within five subdistricts of Segamat, Malaysia.

The survey produced information on family characteristics, housing circumstances, employment, and basic health information. In particular it documented the number of chronic diseases the individual suffers from, whether the individual has high blood pressure, and the contacts with the health system. Our sample included all Malaysian citizens, fifteen to sixty years of age, who participated in the census round.

The median age of the working citizens of Segamat is 38, with a mean age of 37.2 ± 14.3, ranging from 15 to 60 years for both sexes. Males however have a slightly lower median age of 37 years and a mean of 36.7 ± 14.3 years compared to females, which is 39 and 37.7 ± 14.2 years, respectively.  Table 1 further presents some of the sex differences in health and sociodemographic characteristics. The overall proportion of sex approximates that of the Malaysian national census of 2010 [18], whilst the predominant ethnic group is the Malay (~70%). The majority of individuals surveyed were either married (61%) or single (35%). Less than 3% had no education, whilst a significant proportion had either primary (>23%) or secondary (~62%) education. Though 28% reported at least one chronic disease, less than 2% of the population surveyed assessed their health status as either critical or unsatisfactory. Over 73% were uninsured.

The number of chronic diseases is a count data variable; it counts how many separate chronic diseases the individual has at the time of the survey. In order to test the impact of education and work on the number of chronic diseases, we used the negative binomial model. The negative binomial model was applied due to overdispersion observed in our data. To examine the impact of education and work on health we create a dummy variable indicating whether a worker has either completed a diploma or started or finished a degree and define this as tertiary education. This variable “ter” is then made to interact with the various occupation classifications. The coefficient estimates on these interaction terms will tell us if there is an additional impact on chronic disease counts from being tertiary educated in that occupation.

We measure health system contacts by adding together all positive responses to the question “In the past two weeks has the respondent visited any of the following for health reasons?” The categories provided were private hospital, government hospital, private clinic, government clinic, pharmacist, and complementary medical practitioner. It is possible that an individual may visit a particular health provider more than once in the two-week period. Thus, our variable will be a lower bound estimate of contacts with the health system. As with the chronic disease variable, this was treated as a count data variable and was estimated by a Poisson model as the Pearson statistic indicated overdispersion was not a significant problem in this case.

The other key health indicator that we use is a binary variable indicating whether the individual had high blood pressure or not. For this analysis we used a simple probit model. The results from the regression were used to obtain elasticities indicating the percentage change in high blood pressure for a one percentage change in the independent variable at differing education levels, averaged over the estimation sample.

Occupation was classified in 18 categories of job areas including agriculture (agric), business/management (mang), construction (con), education (ed), engineering, technical work/maintenance (eng), food service/hospitality (food), government office (gov), laborer/informal work (lab), manufacturing/factory (man), sales, retail/services (sal), security services (sec), transport/logistics (trans), legal profession & finance & information technology & media, creative design (prof), health services (health), and other (oth).

Education level was classified in 11 categories and coded ordinal as follows: never attended school (1), attended but did not finish primary school (2), finished primary school (3), started high school (4), finished form 3 (5), finished form 5 (6), finished form 6 (7), started college diploma (8), finished college diploma (9), started university (10), and finished university (11). The variable “ter” aggregated educational levels 9, 10, and 11.

## 3. Results and Discussion

**Figure 1:**
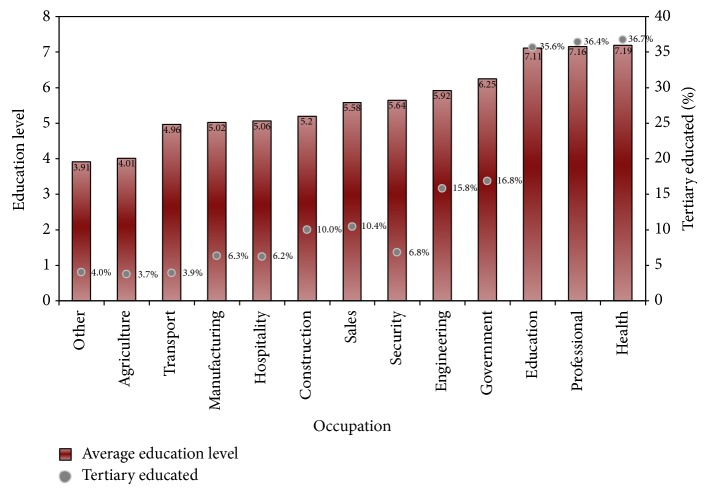
Education levels in occupations.

Summary data on education levels and the percentage of tertiary educated by occupational grouping is illustrated in Figure 1. In agriculture, the average level of education is 4 (started high school) and only 4% of workers are tertiary educated. The health, professional, and education sectors each have an average level of education of 7 (finished high school) and about one-third have tertiary level qualifications.

### 3.1. Chronic Diseases

The results for chronic disease counts are presented in column two of Table 2. The Pearson statistic is highly significant indicating that a negative binomial model should be employed. The results in Table 2 are reported as the incident rate ratios (IRRs); these provide a more meaningful interpretation of the effects. For example, the direct effect of education, as expected, is to lower chronic disease counts as the IRR is lower than one. Thus, a one-year increase in education level reduces chronic disease counts by approximately 3.6%. Age is associated with higher chronic disease counts [16, 19], one year increasing counts by 15% but this impact decreases with age as indicated by the negative squared term. Using the coefficient estimates, rather than IRRs, we can determine that the turning point is approximately 79 years where age and chronic disease develop into a negative relationship. Thus in practice for most individuals chronic disease counts rise with age [19]. Our key interest is the relationship between education level, work, and health. If we first look at the occupation effects we note that workers in government (28%), security (38%), and professional (28%) occupations are associated with higher chronic disease counts than the base (omitted) case of laborers. The interaction terms suggest that being a tertiary educated worker in agriculture, construction, manufacturing, and retail sectors leads to higher chronic disease counts (7%, 8%, 9%, and 5%), though in the latter three cases the coefficients are only marginally significant. These interaction results offset the direct effect of education. Interestingly, in the government sector, which is associated with higher chronic disease counts, tertiary educated workers have significantly lower incidences of chronic disease counts (13%) than other workers in the sector.

Next we examine if the relationships between education, occupation, and chronic disease counts are translated into increased contacts with the health system. We conduct a similar count data regression with number of health system visits as the dependent variable and the results are presented in column three of Table 2. The Pearson statistic produced a chi-squared statistic of 0.21, indicating that overdispersion is not a serious problem in this case, so a Poisson model was used. The direct effect of education is very similar to that seen in the chronic disease counts; a one-unit increase in education years reduces health system visits by 5%. We do not find any evidence of increased usage of the health care system by workers in occupations with higher chronic disease counts (government, security, and professionals). However, it is evident that in two of the four occupations with significantly higher chronic disease counts amongst tertiary educated workers, agriculture and construction, health system visits are significantly higher. In agriculture, visit counts increased by 11.6% and in construction by 16% compared to other workers in these occupations. As above, the interaction effects offset the direct impact of higher education. These findings extend our knowledge of the links between education, occupation, and their collective potential impact on health [10, 12].

### 3.2. High Blood Pressure (HBP)

**Figure 2:**
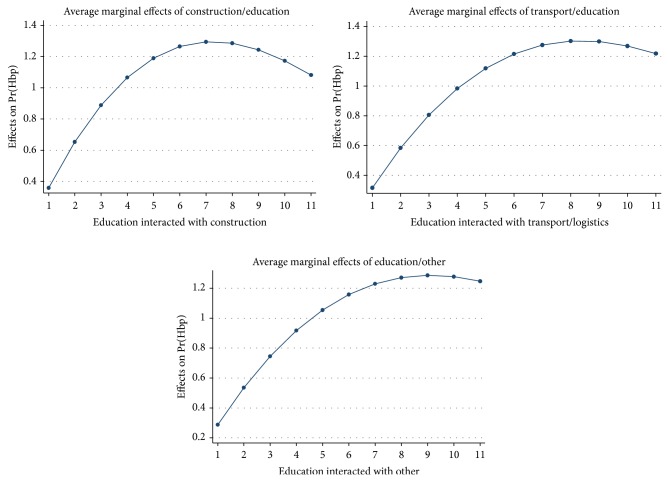
Education level and high blood pressure in selected industries (elasticities at means).

The estimated coefficients from the HBP model are presented in Table 3. In this case we made the occupational indicators interact with the education variable identifying all levels of education. As we have a binary dependent variable, interaction variables of occupation and tertiary education “ter” were in many cases not able to be estimated as they perfectly predicted either success or failure. The significant factors increasing the likelihood of HBP are age, with a turning point at 75 yrs, gender, people from indigenous ethnic groups (Orang Asli), and all districts other than Sungai Segamat, the omitted category. Working in construction, transport, and other occupations appears to be associated with lower incidence of HBP than laboring, the omitted category. However, for more educated workers in these occupations there is a significantly increased likelihood of HBP. To provide a better picture of the effect of education in these occupations on HBP we calculate the elasticities of HBP with respect to education. This is done for the eleven education levels and plotted on graphs in Figure 2. These elasticities are the average marginal effects (AME), a population averaged marginal effect, or the mean marginal effect for the population; this is preferred to the elasticity evaluated at the means of all covariates. The values obtained are the percentage change in HBP for a one percentage change in education level. In the construction and transport occupations the elasticity increases in value up to the postschool level and then declines but remains higher than the AME for lower educated workers. In the “other” occupations category the elasticity value remains higher at the tertiary level. All tertiary level elasticities are greater than one (elastic) whereas workers in these occupations who have not completed secondary school have elasticities substantially below one (inelastic). Finally, in Table 4 we document the instances of health system visits by whether workers have HBP or not. It is clear that multiple visits are much more likely to occur for those with HBP. In unreported analysis, we included HBP as an extra covariate in the Poisson regression with health system contacts as the dependent variable. The IRR coefficient on HBP was 3.151 and was significant at the 1% level, suggesting that the conditional effect of HBP is to increase contacts by 215%.

## 4. Conclusions

This study identified that education is in general correlated with better health outcomes, as much previous research has found. However, in certain occupations, higher education levels were associated with adverse health outcomes. This occurred in occupations with fewer tertiary educated people and where education levels are generally lower. In agriculture and construction, tertiary educated individuals are more likely to have higher counts of chronic diseases and this translates into more contacts with the health system. Construction, transport, and “other” occupations are, in general, associated with lower blood pressure incidence than most other occupations. Yet, the propensity to suffer increased risk of high blood pressure increases with education levels in these occupations. High blood pressure also increases the likelihood of contact with the health system.

The agriculture industry deserves particular consideration; it is the major private sector employer in the rural district where the data was collected and agriculture is viewed as a key sector in rural development in Malaysia. Tertiary educated individuals within the agriculture industry are less likely to be working with other educated individuals. Thus, they may be more exposed to work that is less mentally demanding and receive less stimulation in the work environment than in other occupations. Nevertheless, these workers are important for the productivity and sustainability of the sector. The presence of increased health issues for tertiary educated workers is a concern.

Two major policy implications can be derived from these results. Policy makers associated with regional and rural development should consider programs to increase networking in the agriculture sector to help educated individuals develop more stimulating contacts with other educated workers in the sector. In addition, the sector itself should be encouraged to help. For example, in larger agricultural organisations there could be scope to implement or enhance job enrichment plans. Secondly, workers in the District Health Services in Malaysia's rural regions should be trained to understand the health issues that educated workers may face later in life if current health problems are evident. This may mean some revision in the focus of training away from the traditional key groups such as the poor and those with low status work. Moreover, improved health outcomes for educated workers can translate into increased productivity in the rural economy and this could well outweigh the investment cost in training.

## Figures and Tables

**Table 1:** Health and sociodemographic characteristics of working population (citizens) by sex differences in Segamat, Malaysia.

Variable	Female		Male		Total	
	Number	%	Number	%	Number	Col. %
Residents sex	10,981	51.9	10,167	48.1	21,148	N/A
Age categories						
15–24 y	2,818	49.8	2,836	50.2	5,654	26.7
25–38 y	2,608	50.7	2,536	49.3	5,144	24.3
39–50 y	2,822	53.8	2,425	46.2	5,247	24.8
51–60 y	2,733	53.6	2,370	46.4	5,103	24.1
Ethnicity						
Malay	7,378	51.9	6,838	48.1	14,216	69.2
Chinese	1,925	53.4	1,678	46.6	3,603	17.5
Indian	1,224	54.1	1,038	45.9	2,262	11.0
Indigenous	234	51.5	220	48.5	454	2.2
Marital status						
Single	3,179	43.5	4,134	56.5	7,313	34.5
Married	7,019	54.6	5,829	45.4	12,848	60.8
Separated/living apart	61	60.4	40	39.6	101	0.5
Divorced	285	85.3	49	14.7	334	1.6
Widowed	429	79.7	109	20.3	538	2.5
Other	8	57.1	6	42.9	14	0.1
Education level						
Never attended school	361	65.9	187	34.1	548	2.8
Attended but did not finish primary school	570	58.3	407	41.7	977	5.0
Finished primary school	2,023	57.0	1,526	43.0	3,549	18.2
Started high school	651	46.4	752	53.6	1,403	7.2
Finished form 3	1,498	48.5	1,591	51.5	3,089	15.8
Finished form 5	3,456	49.1	3,577	50.9	7,033	36.1
Finished form 6	325	62.6	194	37.4	519	2.7
Started college (diploma)	220	51.4	208	48.6	428	2.2
Finished college (diploma)	457	50.8	443	49.2	900	4.6
Started university (diploma)	273	64.2	152	35.8	425	2.2
Finished university	355	56.6	272	43.4	627	3.2
Self-assessed health status						
Very good	3,555	51.2	3,391	48.8	6,946	32.8
Good	6,249	51.9	5,797	48.1	12,046	57.0
Satisfactory	990	55.1	806	44.9	1,796	8.4
Unsatisfactory	172	51.5	162	48.5	334	1.6
Critical	8	61.5	5	38.5	13	0.1
No response	7	53.9	6	46.2	13	0.1
Type of health insurance						
Company	388	31.9	830	68.1	1,218	5.8
Self	2,036	50.0	2,038	50.0	4,074	19.3
None	8,416	54.1	7,152	45.9	15,568	73.6
Other	141	49.0	147	51.0	288	1.3
Chronic disease burden						
No chronic disease	7,690	50.56	7,519	49.44	15,209	71.9
1 chronic disease	2,173	53.77	1,868	46.23	4,041	19.1
2 chronic diseases	755	58.94	526	41.06	1,281	6.1
3 chronic diseases	246	58.85	172	41.15	418	2.0
4 chronic diseases	80	59.26	55	40.74	135	0.6
≥5 chronic diseases	37	57.81	27	42.19	64	0.3
Area of occupation						
Agriculture	309	20.5	1,198	79.5	1,507	18.0
Business/management	13	52.0	12	48.0	25	0.3
Construction	11	3.3	319	96.7	330	3.9
Education	966	54.0	823	46.0	1,789	21.3
Engineering, technology/maintenance	22	5.4	386	94.6	408	4.9
Finance	60	52.6	54	47.4	114	1.4
Food service/hospitality	214	59.3	147	40.7	361	4.3
Government office	115	26.0	328	74.0	443	5.3
Information technology	14	26.4	39	73.6	53	0.6
Laborer/informal work	152	31.9	325	68.1	477	5.7
Legal profession	10	58.8	7	41.2	17	0.2
Manufacturing/factory	163	38.6	259	61.4	422	5.0
Media/creative design	12	21.4	44	78.6	56	0.7
Other	60	16.9	295	83.1	355	4.2
Sales retail/services	430	52.1	395	47.9	825	9.8
Health services	176	69.3	78	30.7	254	3.0
Security services	48	10.6	405	89.4	453	5.4
Transport/logistics	6	1.2	498	98.8	504	6.0
Subdistrict						
Bekok	1,372	49.8	1,382	50.2	2,754	16.5
Chaah	2,452	53.8	2,106	46.2	4,558	27.2
Gemereh	1,222	53.1	1,078	46.9	2,300	13.7
Jabi	1,879	52.9	1,674	47.1	3,553	21.2
Sungai Segamat	1,835	51.3	1,741	48.7	3,576	21.4

**Table 2:** Chronic disease and hospital visits.

Variables	Number of chronic diseases (negative binomial model)	Health system contacts (Poisson model)
Education	0.964^***^ (0.0133)	0.949^**^ (0.0245)
Residents age	1.153^***^ (0.0095)	1.089^***^ (0.0144)
Residents age squared	0.999^***^ (7.91*e* − 05)	0.999^***^ (0.000125)
Sex (omitted case: male)		
Female	1.139^***^ (0.0486)	1.286^***^ (0.0996)
Marital status (omitted case: single)		
Married	0.945 (0.0605)	1.057 (0.121)
Divorced	1.084 (0.135)	0.963 (0.231)
Widowed	0.998 (0.1000)	0.873 (0.169)
Separated	1.125 (0.261)	0.808 (0.415)
Household residents	0.997 (0.0083)	0.956^***^ (0.016)
Ethnicity (omitted case: Malay)		
Chinese	1.187^***^ (0.0618)	1.045 (0.101)
Indian	1.255^***^ (0.0917)	0.993 (0.133)
Indigenous	1.206 (0.140)	1.502^**^ (0.250)
Other ethnicities	0.999 (0.256)	0.735 (0.372)
Self-insurance	1.113^**^ (0.054)	1.112 (0.101)
Occupation (omitted case: labourer)		
agric	0.998 (0.077)	0.951 (0.129)
mang	1.803^*^ (0.616)	1.999 (1.185)
con	0.879 (0.118)	0.851 (0.216)
edu	1.102 (0.0968)	1.085 (0.169)
eng	1.045 (0.137)	0.973 (0.231)
food	1.088 (0.118)	0.865 (0.176)
gov	1.282^**^ (0.145)	1.340 (0.270)
man	1.092 (0.121)	0.776 (0.176)
oth	1.164 (0.116)	0.723^*^ (0.140)
sal	1.024 (0.105)	0.784 (0.150)
health	0.936 (0.162)	0.939 (0.296)
sec	1.380^***^ (0.144)	1.093 (0.212)
tran	0.997 (0.106)	1.174 (0.214)
prof	1.283^*^ (0.193)	1.147 (0.332)
Interactions: occupation and tertiary education		
Agric^*^ter	1.073^***^ (0.533)	1.116^***^ (0.045)
Mang^*^ter	0.910 (0.093)	0.207 (0.042)
Con^*^ter	1.083^*^ (0.048)	1.160^***^ (0.068)
Edu^*^ter	1.000 (0.112)	0.993 (0.207)
Eng^*^ter	1.008 (0.038)	1.040 (0.061)
Food^*^ter	0.965 (0.072)	0.209 (0.035)
Gov^*^ter	0.867^***^ (0.048)	0.842 (0.093)
Man^*^ter	1.089^*^ (0.048)	0.220 (0.358)
Oth^*^ter	0.971 (0.059)	1.049 (0.102)
Sal^*^ter	1.055^*^ (0.035)	0.926 (0.102)
Health^*^ter	1.022 (0.031)	0.969 (0.061)
Sec^*^ter	0.906 (0.066)	1.058 (0.078)
Tran^*^ter	0.932 (0.100)	0.207 (44.945)
Prof^*^ter	1.033 (0.028)	1.021 (0.052)
Subdistrict (omitted case: Sungai Segamat)		
Bekok	2.091^***^ (0.125)	2.946^***^ (0.325)
Gemereh	1.899^***^ (0.120)	1.251^*^ (0.171)
Chaah	1.376^***^ (0.113)	1.831^***^ (0.273)
Jabi	1.016 (0.070)	1.263^*^ (0.165)
Other (unlisted subdistrict)	1.179^***^ (0.068)	1.388^***^ (0.155)
Constant	0.004^***^ (0.0009)	0.006^***^ (0.002)
Observations	8,849	8,849

Standard errors in parentheses, ^***^ *P* < 0.01, ^**^ *P* < 0.05, and ^*^ *P* < 0.1, lnalpha, chi-squared(1) = 40.00 (0.00).

Pearson Prob chi2(8,800) = 0.00 (chronic disease), chi2(8,800) = 0.21 (doctor visits).

**Table 3:** Probability of high blood pressure (probit).

Variables	High blood pressure
Education	−0.061 (0.055)
Residents age	0.135^***^ (0.013)
Residents age squared	−0.0009^***^ (0.0001)
Female	0.138^***^ (0.051)
Married	0.103 (0.085)
Divorced	−0.028 (0.160)
Widowed	0.077 (0.126)
Separated	0.211 (0.271)
Household residents	−0.019^*^ (0.010)
Chinese	0.007 (0.063)
Indian	0.082 (0.089)
Indigenous	0.377^***^ (0.161)
Other ethnicities	0.070 (0.291)
Self-insurance	−0.008 (0.057)
agric	−0.258 (0.233)
mang	0.293 (1.139)
con	−1.127^***^ (0.411)
edu	0.086 (0.252)
eng	−0.317 (0.409)
food	0.181 (0.337)
gov	0.293 (0.403)
man	−0.336 (0.358)
oth	−0.596^**^ (0.279)
sal	−0.051 (0.305)
health	−0.283 (0.514)
sec	0.258 (0.388)
tran	−0.799^**^ (0.380)
prof	−0.047 (0.418)
agriced	0.079 (0.059)
manged	−0.094 (0.149)
coned	0.219^***^ (0.081)
edued	0.027 (0.058)
enged	0.080 (0.079)
fooded	0.016 (0.078)
goved	−0.014 (0.078)
maned	0.120 (0.079)
othed	0.146^**^ (0.068)
saled	0.053 (0.068)
healthed	0.085 (0.082)
seced	0.004 (0.080)
traned	0.191^**^ (0.084)
profed	0.047 (0.073)
Bekok	0.230^***^ (0.076)
Gemereh	0.196^***^ (0.077)
Chaah	0.224^***^ (0.091)
Jabi	0.133^*^ (0.078)
Other (unlisted subdistrict)	0.164^***^ (0.063)
Constant	−5.530^***^ (0.428)
Pseudo *R* ^2^	0.19
Observations	8,849

Standard errors in parentheses ^***^ *P* < 0.01, ^**^ *P* < 0.05, and ^*^ *P* < 0.1.

**Table 4:** High blood pressure (HBP) and health system contacts.

Number of visits	HBP	Other	% HBP
0	599	7,472	8.0
1	321	560	57.3
2	16	30	53.3
3	3	4	75%
